# Health Preferences in Transition: Differences from Pandemic to Post-Pandemic in Valuation of COVID-19 and RSV Illness in Children and Adults

**DOI:** 10.3390/children12020181

**Published:** 2025-01-31

**Authors:** Kerra R. Mercon, Angela M. Rose, Christopher J. Cadham, Acham Gebremariam, Jamison Pike, Eve Wittenberg, Lisa A. Prosser

**Affiliations:** 1 Susan B. Meister Child Health Evaluation and Research Center, University of Michigan, Ann Arbor, MI 48109, USA; kmercon@umich.edu (K.R.M.); angmrose@umich.edu (A.M.R.); agmariam@umich.edu (A.G.); 2 Department of Health Management and Policy, School of Public Health, University of Michigan, Ann Arbor, MI 48109, USA; ccadham@umich.edu; 3 Centers for Disease Control and Prevention, Atlanta, GA 30333, USA; kqv1@cdc.gov; 4 Center for Health Decision Science, Harvard T. H. Chan School of Public Health, Boston, MA 02115, USA; ewittenb@hsph.harvard.edu

**Keywords:** preference valuation, COVID-19, RSV, pediatrics, time trade-off, QALYs

## Abstract

**Objective:** This study aimed to measure changes in preferences regarding health-related quality of life associated with COVID-19 and RSV illness in children and adults from 2021 (during the COVID-19 pandemic) to 2023 (post-pandemic). **Methods:** A stated-preference survey elicited time trade-off (TTO) values from US adults in spring 2021 (*n* = 1014) and summer 2023 (*n* = 1186). Respondents were asked to indicate how much time they would hypothetically be willing to trade from the end of their life to avoid the effects of varying severities of COVID-19 and RSV illness for: (1) children; (2) parents of an ill child (family spillover); and (3) adults. Attitudes relating to COVID-19 vaccination and data on experience with COVID-19 or RSV illness were also collected. The primary outcome measure was the loss in quality-adjusted life years (QALYs). Changes in preferences over the time period from 2021 to 2023 were evaluated using regression analysis. **Results:** QALY losses increased with disease severity and were highest for Long COVID. Across all COVID-19 and RSV health states, QALY losses associated with child health states were higher than family spillover or adult health states. In the regression analysis, QALY losses reported in the 2023 survey were significantly lower than 2021 QALY losses for COVID-19, but not RSV. **Conclusions:** Preferences may change over time in a pandemic context and therefore, economic analyses of pandemic interventions should consider the timeframe of health preference data collection to determine whether they are suitable to include in an economic evaluation. Even with the impacts on health-related quality of life attenuated over time, childhood illnesses still had a measurable impact on caregivers’ quality of life.

## 1. Introduction

Coronavirus disease 2019 (COVID-19) and respiratory syncytial virus (RSV) cause significant morbidity in the United States [1,2]. COVID-19 illness causes a large spectrum of symptoms and symptom severity and can result in complications requiring hospitalization and/or longer-term sequelae, including over 500 hospitalizations per 100,000 people during the 2021–2022 season [3]. Due to concern about disease transmission, COVID-19 patients were often required to isolate from others for up to 10 days after symptom onset [4,5]. RSV is especially problematic for the very young and older adults. RSV represents the most common cause of hospitalization for infants, and up to 10,000 deaths are attributable to RSV in older adults each year [1,6]. Experiencing COVID-19 or RSV symptoms can reduce health-related quality of life (HRQoL). Given the burden of these conditions, new vaccines have been introduced within the last 3 years for prevention of both COVID-19 and RSV illnesses [7,8,9].

The value of prevention and treatment programs such as widespread vaccination can be assessed through economic evaluations where the incremental costs of an intervention and the accrued health benefits of the intervention are calculated [10]. In cost-effectiveness analysis, a type of economic evaluation, the measure of HRQoL benefit is typically the quality-adjusted life year (QALY) [11]. Efforts to understand the HRQoL effects of severe COVID-19 illness and hospitalization with COVID-19 on quality of life have been ongoing globally [12,13,14,15,16,17,18,19,20,21,22,23,24,25,26,27,28]; however, many of these studies have not reported results in measures that can be used to generate QALYs and most have been conducted outside of the United States (US). The primary focus of most existing studies of HRQoL related to COVID-19 have focused on severe illness rather than non-hospitalized illness due to COVID-19. As COVID-19 moves into an endemic state, understanding the effects of non-hospitalized COVID-19 on HRQoL has become increasingly important. Similarly, cases of RSV continue to lead to mild and severe illness in children and adults. Several studies have reported HRQoL estimates for RSV patients, but these were all conducted in countries outside of the USA (Spain [29,30], the UK [31], the Netherlands [32], and Finland [33]). Measuring the impact of COVID-19 and RSV illness using QALYs is essential for understanding the burden of disease and to support cost-effectiveness analyses of new vaccines for these illnesses.

The objective of this study was to build on this previous work and evaluate the impact of two respiratory diseases with a large public health impact, COVID-19 and RSV illness, on HRQoL of children, parents, and adults in the US setting, for varying severities of illness. This survey was originally developed to assess preferences for RSV health states, but the COVID-19 pandemic was declared before the survey was distributed. Due to concern about how the COVID-19 pandemic would affect respondents’ preferences, we delayed fielding the survey, and added questions relating to COVID-19 health states. The survey was first administered during the COVID-19 pandemic in the spring of 2021. To assess changes in COVID-19 and RSV preferences that occurred during and after the COVID-19 pandemic, the survey was administered a second time after the COVID-19 public health emergency ended in April 2023 [34]. This study addresses the question: what was the impact of COVID-19 and RSV illness on the HRQoL of children, parents, and adults in the United States in 2021 and 2023? Our hypothesis was that COVID-19 preferences would change over time as the pandemic abated and that RSV preferences would remain static. Our study adds to the existing literature by providing new data from direct ascertainment of QALYs lost, elicited using the time trade-off approach, to quantify the impact of COVID-19 or RSV illness on the HRQoL of children, their parents, and adults and explore differences over time.

## 2. Materials and Methods

An online, stated-preference survey was developed that used direct valuation to elicit quantitative measurements of maximum time trade-off (TTO) amounts. The TTO approach asked respondents to hypothetically give up a number of days from the end of their life to avoid an episode of COVID-19 or RSV illness. This TTO approach has been found to be reliable [35] and valid [36] and has been used previously to elicit preferences for childhood and adult health states [10,37,38] and family spillover effects [39].

### 2.1. Study Participants

The survey was administered twice to nationally representative panels. It was originally administered in spring 2021 to respondents in the NORC at the University of Chicago AmeriSpeak panel. AmeriSpeak is a probability-based panel designed to be nationally representative of the US adult population [40]. Randomly selected households are sampled using area probability and address-based sampling, with a known, non-zero probability of selection from the NORC National Sample Frame. The panel provides sample coverage of approximately 97% of the US household population, with a 22% recruitment rate and 78% panel retention rate. This survey had a completion rate of 30% for a cumulative response rate of 5.2%.

To examine COVID-19 preferences at the declaration of the end of the pandemic, the survey was again administered in summer 2023 to a separate national sample of respondents from opt-in panels contracted through Qualtrics (Seattle, USA). Quotas were used to achieve nationally representative proportions of respondents by age, gender, race/ethnicity, and household income. Due to the sampling methodology, a response rate could not be calculated for this sample. All elements of the survey remained the same between the 2021 and 2023 survey administrations, except for additional demographic questions in the 2023 survey administration.

### 2.2. Survey Design and Development

The survey included four sections: an introduction that explained the TTO task, TTO practice questions, TTO health state valuations, and questions concerning the respondent’s health and attitudes toward COVID-19.

Health state descriptions for the TTO tasks were developed following a review of the literature [5,41,42,43,44,45,46] and in consultation with a convenience sample of recovered patients and medical experts on COVID-19 and RSV illness. Health states included information on disease severity, duration, and treatment. For COVID-19, the health states also included information about quarantine, isolation, and uncertainty related to the COVID-19 disease progression (see health state descriptions in Supplementary Table S1). This study was conducted to provide measures of the change in HRQOL suitable for inclusion in an economic evaluation, and health states were selected to reflect those typically considered for economic analysis of prevention or treatment of respiratory illness [47,48,49]. They included four levels of COVID-19 severity: (1) outpatient, (2) hospitalized, (3) hospitalization for multisystem inflammatory syndrome in children (MIS-C) or hospitalization including intensive care unit (ICU) stay in adults, and (4) Long COVID; and two levels of RSV severity: (1) outpatient, and (2) hospitalized (Supplementary Table S1). Iterative versions of the survey were pretested in both paper and online formats (*n* = 16).

Several intentional departures from conventional methods were incorporated into the design of the health state descriptions for COVID-19 illness. First, each health state was labeled to indicate whether the scenario was related to COVID-19 or RSV illness, to allow respondents to consider their knowledge of the illnesses beyond the survey descriptions. The descriptions of each COVID-19 health state introduced uncertainty about future progression of the disease (e.g., whether the respondent could expect to improve or worsen). The descriptions also included effects beyond the individual, describing concerns about transmitting the illness to others. Effects on domains not typically considered when measuring HRQoL, such as isolation from family and friends, were also described (Supplementary Table S1).

### 2.3. Survey Instrument

#### 2.3.1. Health State Valuation

Respondents completed practice questions to gain familiarity with the TTO valuation task. Then, each respondent evaluated a subset of the four health states in three question frames, based on methods used in prior studies [37,38,50]: (1) adult health state (≥18 years of age; asking the respondent to imagine how they would feel if they had the illness; Supplementary Figure S1), (2) child health state (<18 years of age; asking the respondent to consider how *their child* would feel if *their child* had the illness), and (3) as a parent of an ill child (“spillover frame”, asking the respondent to imagine how *they* would feel if *their child* had the illness). Respondents were asked how much time they would be willing to trade from the end of their life to avoid the described health state. The TTO task used dichotomous-choice double-bounded bids followed by an open-ended question [38]. Respondents answered questions using an initial bid (selected amounts of time to trade off), followed by a second question with a higher or lower bid based on the response to the initial bid (Supplementary Table S2). For example, respondents were first asked if they would be willing to give up 2 days from the end of their life to avoid outpatient COVID-19 or RSV illness. If the respondent selected yes, the bid increased to 4 days. If the respondent selected no, the bid decreased to 1 day. Respondents were randomized to one of four groups of bid vectors for each health state (e.g., outpatient COVID-19/RSV). All respondents were then asked to state the maximum amount of time they would be willing to give up from the end of their life to avoid the effects of the illness. This pattern was followed for all 3 frames (adult, child, spillover). Each respondent received personalized information on remaining life years based on the respondent’s age and national estimates of life expectancy in the United States [51].

#### 2.3.2. Health Questions and Demographics

The final section of the survey contained questions concerning the respondent’s overall health, previous experience with COVID-19 or RSV illness, COVID-19 vaccination status/intent, and opinions about the risk of COVID-19 [52,53].

The survey also asked about the difficulty of the questions, and respondents were given the opportunity to enter free-text survey feedback. Finally, respondents indicated if they were thinking of a child of a certain age when answering the TTO questions.

### 2.4. Survey Administration

This survey was administered to cross-sectional samples from two panels. The 2021 survey was pilot tested online in March 2021 (n = 105) and was then administered in April and May 2021. The median survey completion time was 10 min and respondents received a USD 3 incentive for completion, provided by the survey vendor. The 2023 survey was pilot tested online in June 2023 (n = 100) and administered in June and July 2023. The median survey completion time was 10.3 min and respondents received incentive points for completion, which could be redeemed with the vendor for gift cards, charitable donations, or other incentives.

This study was approved as exempt human-subject research by the University of Michigan Medical School Institutional Review Board and was performed in accordance with the ethical standards in the 1964 Declaration of Helsinki and its later amendments. This activity was reviewed by CDC and was conducted consistent with applicable federal law and CDC policy. (See e.g., 45 C.F.R. part 46, 21 C.F.R. part 56; 42 U.S.C. §241(d); 5 U.S.C. §552a; 44 U.S.C. §3501 et seq.)

### 2.5. Data Analysis

Differences between the 2021 and 2023 samples in terms of sociodemographic factors and experiences and attitudes associated with COVID-19 and RSV illness were compared using the chi-square test for categorical covariates and two-sample t-tests to compare the numerical variables.

The main outcome measure was mean QALY loss, derived by dividing the maximum TTO amount by life expectancy at the individual respondent level [37]. Descriptive statistics including mean, median, and bootstrapped 95% confidence intervals were calculated to describe QALY losses for each health state question frame (child, spillover, adult). Statistical analyses were conducted using STATA/SE v15 statistical software (College Station, TX, USA). Respondents were excluded from the primary analysis if they responded that they did not understand the task, entered a protest comment, or gave a poor-quality TTO response (Supplemental Table S3).

Secondary analyses were conducted on the 2021 survey administration data and excluded respondents who answered less than 50% of the 2021 TTO questions or who stated they would be willing to give up the same non-zero amount of time for each TTO question [54]. Additional analyses examined adjustments for expected life expectancy, discounting future health and adjusting for worse quality of life at the end of life [55]. Stratified analyses examined differences in QALYs lost by concern about COVID-19 or intention to receive the COVID-19 vaccination.

Multivariate analyses using a two-part beta regression model [56] were conducted to evaluate any associations between QALY losses and sociodemographic factors, experience with COVID-19 or RSV illness, attitudes about COVID-19, and survey administration year (2021 compared to 2023). The dependent variable was QALY loss. Independent variables were health states (outpatient, hospitalization, MIS-C/ICU, Long COVID), sociodemographic variables (gender, age, race/ethnicity, education, marital status, income, region, and households with children), health experiences and attitudes (overall health, experience with COVID-19, experience with RSV, COVID-19 vaccine intent, level of concern about COVID-19), and survey administration year (2021 or 2023). COVID-19 vaccination status/intent was assessed with the question: “What have you done or are you planning to do regarding the COVID-19 vaccine?” (Table 1). Opinions about the risk of COVID-19 were assessed according to level of agreement with the statement: “COVID-19 is not as big of a problem as the media suggests”. Analyses were adjusted for clustering at the respondent level to account for responses to multiple health states and frames. Separate models were analyzed for each of the three frames (child, spillover, adult).

Finally, a Kolmogorov–Smirnov test was conducted to compare the outcome distributions of QALY losses for the 2021 and 2023 survey administrations for each health state and determine whether they differed significantly [57].

## 3. Results

### 3.1. Respondent Demographics and Attitudes

Twenty-nine percent of the 2021 sample reported they had family experience with COVID-19 illness, compared with 60% in the 2023 sample (Table 1; Supplementary Table S4). Reported family experience with RSV was far less common in both samples (2021: 6%; 2023: 9%). More than 70% of both the 2021 and 2023 survey administration respondents had either received the primary series of the COVID-19 vaccine or intended to receive it soon. In 2021, 33% of respondents agreed or strongly agreed with the statement: “COVID-19 is not as big of a problem as the media suggests”, compared with 45% of the 2023 survey administration respondents.

### 3.2. Health-Related Quality of Life Measures

QALY losses increased with health state severity and were highest for Long COVID. Across all COVID-19 and RSV health states, median QALY losses in the 2021 sample were generally higher in the child frame: 0.001 for outpatients, 0.005 for hospitalization, 0.011 for MIS-C, 0.019 for Long COVID (Table 2). QALY losses for adults were 0.001 for outpatient COVID-19 illness, 0.003 for COVID-19 hospitalization, 0.004 for hospitalization requiring ICU care, and 0.008 for Long COVID (Table 2). QALY losses associated with RSV health states were lower than losses for COVID-19 health states. Quality-adjusted life days (QALDs) lost associated with COVID-19 and RSV in the 2021 survey can be found in Supplementary Tables S5 and S6. QALYs lost from the 2023 survey can be found in Supplementary Table S7.

### 3.3. Secondary and Stratified Analyses

The results of secondary analyses incorporating additional exclusion rules (Supplementary Tables S8 and S9) were comparable to the primary analysis. Secondary analyses that used alternative approaches to calculate mean QALY losses yielded lower results compared with the primary analysis (Supplementary Tables S8 and S9). In stratified analyses, QALYs lost associated with COVID-19 were generally higher in those more concerned about COVID-19 and who intended to receive the COVID-19 vaccine (Supplemental Table S10).

### 3.4. Multivariate Regression Analysis

In the regression analysis, the 2023 survey administration year was associated with lower QALY losses for both the child and adult frames for COVID-19 illness but not for family spillover effects from COVID-19 or any RSV frame (Table 3 and Table 4, Supplementary Tables S11 and S12). Less severe health states were associated with lower QALY losses across frames for both COVID-19 and RSV. For COVID-19 illness in the child and spillover frames, “no COVID-19 vaccination intent” and “less concern about COVID-19” were also associated with lower QALY losses. Additional regression analyses controlling for sociodemographic variables only can be found in Supplementary Tables S13 and S14. The results of the Kolmogorov–Smirnov test also demonstrated that QALY losses were the same or higher in the 2021 survey administration for all child and adult health states (Supplementary Table S15).

## 4. Discussion

This study estimated losses in HRQoL (QALY losses) resulting from COVID-19 and RSV illness in children, parents, and adults. We found that respondents were willing to trade more time from the end of their life to avoid severe symptoms and Long COVID, compared to less severe states. QALY losses were higher for an affected child than an affected adult across all health states, with family spillover QALY losses typically between these two sets of values. Our study adds new data from direct ascertainment of QALYs lost through the TTO approach to quantify the impact of COVID-19 or RSV infection on the HRQoL of children, their parents, and adultsand explore differences over time. To our knowledge, no other study has applied a direct-elicitation TTO approach to generate QALYs for COVID-19 and RSV.

Compared with the present study, other studies from the United States have reported variable QALY losses for COVID-19 illness. A 2023 study by Tak measured HRQoL due to COVID-19 illness using the EQ-5D-5L, with healthcare utilization as a proxy for symptom severity [28]. Applying the durations of illnesses used in our study (14 days for outpatient illness and 21 days for hospitalized COVID-19), QALYs lost in the Tak study were 0.019 for outpatient and 0.023 for hospitalization. These findings were similar to the mean results from our 2021 survey administration for outpatient illness but lower than our results for hospitalized illness. A study by Sun et al. measured HRQoL using the EQ-5D-5L 3 days after testing positive for symptoms [27]. QALYs lost in Sun et al.’s study were lower than those in our 2021 survey administration (0.007 vs. 0.019, respectively). Differences in survey timing and study setting or between the direct-elicitation TTO format and the EQ-5D may explain this variance.

For RSV illness, our results were typically higher than the losses reported in the literature. One prospective study by Mao et al. conducted across four European countries between 2017–2019 found that QALY loss for infants was 0.006 due to outpatient care for RSV and 0.010 for hospitalized RSV, compared with 0.045 and 0.073, respectively, in the 2021 survey administration of the present study [58]. Similarly, another study conducted in the UK estimated a much higher mean QALY loss per acute RSV episode, ranging from 0.0015 for non-healthcare-seeking patients over 5 years old to 0.00195 for patients greater than 5 [31]. These studies were fielded between 2017–2020, before the start of the COVID-19 pandemic, which could be associated with lower perceived QALY loss (pre-pandemic) for respondents, as the pandemic may have increased concerns about respiratory illnesses similar to COVID-19.

In the regression analysis, QALY losses for COVID-19 in children and adults were significantly lower in the 2023 sample as compared to the 2021 sample. There were no significant differences in QALY losses for the RSV health states between the two survey administrations except when all three frames were considered together. This suggests, in line with our hypothesis, that as the severity of the COVID-19 pandemic attenuated, respondents perceived COVID-19 illness to have less of an impact on HRQoL but opinions about RSV illness remained unchanged. The results of the Kolmogorov–Smirnov test support this finding; the 2021 distribution typically contained the same or larger values than the 2023 sample (Supplementary Table S15). As 45% of the 2023 sample indicated that they agreed that COVID-19 was not as big of a concern as the media suggests, compared with 33% in the 2021 sample, decreased concern about the risks and consequences of COVID-19 illness may have resulted in lower values of TTO in the 2023 sample. This finding highlights the need for economic analyses of pandemic interventions to consider the context and timing of health preferences for suitability to include in an economic evaluation, given changes in epidemiology and the severity of disease over time.

The COVID-19 pandemic also served to highlight several methodologic considerations regarding the elicitation of health utility values, with broader implications for the design of health state descriptions. In this study, we incorporated uncertainty into the health state descriptions to reflect the highly uncertain future trajectory of COVID-19 illness. The conventional approach to developing scenario descriptions would seek to reduce uncertain descriptors to reduce variability in respondents’ interpretation [59,60]. However, in this context, concerns regarding future states of health, worries about transmission to others, and the potential effects of isolation, especially for pediatric patients, were likely to represent important determinants of quality of life; excluding these aspects from a described scenario could result in an incorrect valuation of the health state. Although downstream health effects (such as hospitalization or death) would be included in the later stages of an economic evaluation model, we argue that the worry about these potential long-term sequelae is part of the current health state and should enter separately into the valuation framework.

The pandemic context also highlighted the constraints of cost-effectiveness analysis as a valuation framework, prompting consideration of alternative frameworks for valuing health interventions, such as cost–benefit analysis or other elements of value, as suggested by the ISPOR professional society for health economics and outcomes research Special Taskforce [61]. First, restricting the measure of benefit to only HRQoL is unlikely to capture the full value of an intervention that may have impacts on broader aspects of well-being, such as concern about transmission to another or the impact of isolation. In addition, especially for children, isolation can have long-term impacts beyond the health sector like educational or developmental outcomes [62,63].

Family spillover effects are increasingly being included in health economic evaluations to capture the full burden of illness on households [64]. While the impact of child illness on parents’ quality of life has been reported for chronic conditions [65,66], limited research has examined how parents’ quality of life is affected by acute child illness, including COVID-19 and RSV. One study found that caregivers of adult COVID-19 patients had a greater decrease in HRQoL than the COVID-19 patients themselves [26]. Additionally, Mao et al. reported that for caregivers, QALY losses were 0.0005 and 0.003 for outpatient and hospitalized RSV in their infants, respectively [58]. The present study adds to this literature by estimating spillover effects for children with COVID-19 illness. We found values similar to those stated by Mao for caregiver QALY losses for outpatient illness (0.0001), but much smaller losses for hospitalized illness (0.0004). In the regression analysis of the child spillover frame for COVID illness, higher QALY losses were associated with male gender and lower income. For RSV illness, male gender, lower income, no family experience with RSV/COVID-19, and receiving/intending to receive the COVID-19 vaccine were all associated with increased QALY losses.

Despite extensive research to estimate spillover effects, there is an inherent challenge in directly measuring the child spillover frame. Respondents may not adequately separate their concerns for their child (child frame) from the impact of their child’s condition on their personal quality of life (spillover frame), which could lead to spillover effects also being captured within the child frame [50]. At this stage, family spillover measures should be incorporated into cost-effectiveness analyses primarily as scenario analyses, according to methodological guidelines [10].

Several limitations with the present study should be noted. One of our survey frames included child health valuation, a field that is experiencing rapid advances in methodology yet remains without a consensus on best practice [67,68,69]. In this study, child heath scenarios were valued from the ‘own perspective’ of the adult respondent considering their own child’s health. Using alternative perspectives has been shown to influence the resulting utility values [67,70]. In this application, we elected to use the ‘own perspective’ for consistency, given the inclusion of self-rated health states (for both the illness states and the child spillover states) for which the respondent’s own perspective would be the best practice. Furthermore, we described the health states consistent with the available evidence at the time of the survey. However, with new treatments, increasing immunity in the population, and evolving quarantine and isolation guidelines, these health state descriptions may no longer reflect the experience of many COVID-19 patients. In addition, emerging evidence suggests that Long COVID may have multiple subtypes [71], which would not have been captured in this study. Third, self-report of family experience with RSV or COVID-19 illness is likely inaccurate. For example, 90% of children are infected with RSV by their 2nd birthday [72], yet only 6–9% of respondents reported family experience with RSV. However, family experience with these conditions was inconsistently associated with changes in QALY loss. Finally, survey respondents may have differed from non-respondents regardless of the probability-based sample, due to a cumulative response rate of only 5.2%. However, in the regression analysis, none of the demographic characteristics were significantly associated with QALY loss due to COVID-19 or RSV, across all question frames.

## 5. Conclusions

The perceived impact of COVID-19 illness on health-related quality of life decreased from 2021 to 2023. Preferences may change over time in a pandemic context with changes in experiences with illness, expanded use and availability of vaccines and treatments, and decreasing rates of severe illness; therefore, economic analyses of pandemic interventions should consider the timeframe of health preference data collection to determine if the data are suitable to include in an economic evaluation. From a methodological perspective, eliciting preferences about COVID-19 highlighted limitations of conventional approaches to these methods, including the need to include concerns about transmission to others, isolation, and uncertainty about the course of disease in the health state descriptions. Future research could explore the applicability of the design changes tested here to other disease contexts.

## Figures and Tables

**Table 1:** Respondent characteristics.

	2021 Survey Administration *n* = 1004		2023 Survey Administration *n* = 1186		*p*-Value
	No.	%	No.	%	
**Gender**					0.005
Male	496	49.4	510	43.4	
Female	508	50.6	665	56.6	
**Age**					0.475
Mean age, years	48.2	-	48.8	-	
Range years	18–89	-	18–93	-	
**Race/ethnicity**					0.001
White, non-Hispanic	641	63.8	826	69.7	
Hispanic	176	17.5	123	10.4	
Black, non-Hispanic	103	10.3	130	11.0	
Other/mixed, non-Hispanic	84	8.4	107	9.0	
**Highest level of school completed**					0.001
Some college or less	646	64.3	668	56.4	
Bachelor’s degree or higher	358	35.7	517	43.6	
**Marital status**					0.059
Married/living with a partner	574	57.2	634	53.5	
Divorced/separated/widowed	196	19.5	223	18.8	
Single, never married	234	23.3	329	27.7	
**Income level**					0.001
≤Federal poverty level (FPL)	174	17.3	314	26.5	
>FPL but <3× FPL	410	40.8	367	31.0	
≥3× FPL	420	41.8	504	42.5	
**Children in household**					
Yes	193	19.3	404	34.1	
**Self-reported health status**					0.353
Excellent/Very Good/Good	855	85.2	992	83.7	
Fair/Poor	149	14.8	193	16.3	
**Experience with COVID-19 or RSV illness** ^a^					
COVID-19	299	29.8	722	60.9	0.001
RSV	72	7.2	124	10.5	0.007
**COVID-19 vaccination status/intent ^b^**					0.069
Received/scheduled/intend to receive	711	71.0	882	74.5	
Do not intend to receive vaccine soon/ever	290	29.0	302	25.5	
**Opinion about the risk of COVID-19: “COVID-19 is not as problematic as media presents” ^c^**					0.001
Strongly agree/Agree	324	32.4	535	45.2	
Strongly disagree/Disagree	676	67.6	649	54.8	
**Respondents thinking of a child of specific age**	460	45.6	632	53.3	
Mean age of (theoretical) child, years	9.0	-	4.9	-	
Age of (theoretical) child, ^d^ range, years	0–62	-	0–59		

^a^ Respondents could have experience with more than one illness; percentage does not add to 100%. ^b^ Vaccination plans were assessed with the question: “What have you done or are you planning to do regarding the COVID-19 vaccine?” Answer choices were dichotomized for the regression, with those responding that they had already received, were scheduled to receive, or intended to receive the vaccine compared with those who did not intend to receive the vaccine now, but may in the future, and those who intended never to receive the vaccine. ^c^ Concern about COVID-19 was assessed by asking respondents if they agreed with the statement: “COVID-19 is not as big of a problem as the media suggests”. Those who agreed or strongly agreed were termed “less concerned about COVID” in the regression analysis, while those who disagreed or strongly disagreed were termed “more concerned about COVID”. ^d^ Assessed with the question: “Approximately how old is the child you were thinking about when you were answering these questions?”. FPL—Federal Poverty Level.

**Table 2:** Quality-Adjusted Life Years (QALYs) Lost, 2021 survey administration.

a. COVID-19 Illness				
Variable	Median	5th–95th Percentile	Mean	95% CI *
**QALYs lost due to outpatient COVID-19**				
Child	0.001	0.000–0.667	0.070	0.041–0.106
Spillover	0.000	0.000–0.071	0.036	0.015–0.065
Adult	0.001	0.000–0.046	0.019	0.008–0.033
**QALYs lost due to hospitalized COVID-19 (no ICU)**				
Child	0.005	0.000–1.000	0.092	0.064–0.126
Spillover	0.001	0.000–0.294	0.060	0.037–0.085
Adult	0.003	0.000–0.115	0.045	0.025–0.070
**QALYs lost due to hospitalized COVID-19 with complications**				
*MIS-C (children only)*				
Child	0.011	0.000–0.885	0.102	0.073–0.135
Spillover	0.003	0.000–0.547	0.081	0.052–0.116
*COVID ICU (adults only)*	0.004	0.000–0.167	0.046	0.027–0.068
**QALYs lost due to Long COVID**				
Child	0.019	0.000–0.857	0.110	0.082–0.143
Spillover	0.006	0.000–0.313	0.074	0.051–0.105
Adult	0.008	0.000–0.182	0.056	0.035–0.084
**b. RSV Illness**				
**Variable**				
**QALYs lost due to outpatient RSV**				
Child	0.000	0–0.077	0.045	0.021–0.075
Spillover	0.000	0–0.026	0.025	0.008–0.048
Adult	0.000	0–0.011	0.019	0.005–0.035
**QALYs lost due to hospitalized RSV**				
Child	0.001	0–0.242	0.073	0.044–0.106
Spillover	0.000	0–0.111	0.037	0.021–0.057
Adult	0.001	0–0.063	0.019	0.010–0.032

ICU—intensive care unit;; QALY—quality-adjusted life years. * Bootstrapped.

**Table 3:** Effects of sociodemographic variables, health experiences, attitude variables, and survey administration year on QALY losses, using beta regression, COVID-19.

	Child			Spillover		
Variable	Mean	Bootstrap SD	95% CI	Mean	Bootstrap SD	95% CI
**Survey administration year**						
2021	Ref.	-	-	Ref.	-	-
2023	−0.0084	0.0019	−0.0122, −0.0049 *	−0.0018	0.0017	−0.0049, 0.0016
**Health state**						
Outpatient	−0.0309	0.0029	−0.0370, −0.0256 *	−0.0225	0.0026	−0.0281, −0.0175 *
Hospitalization	−0.0168	0.0026	−0.0223, −0.0119 *	−0.0142	0.0025	−0.0195, −0.0095 *
MIS-C	−0.0057	0.0026	−0.0109, −0.0008 *	−0.0059	0.0023	−0.0107, −0.0015 *
Long COVID	Ref.	-	-	Ref.	-	-
**Gender**						
Male	Ref.	-	-	Ref.	-	-
Female	0.0037	0.0017	0.0005, 0.0072 *	−0.0010	0.0016	−0.0042, 0.0021
**Age**	0.0003	0.0001	0.0002, 0.0004 *	0.0001	0.0001	0.0000, 0.0003 *
**Education**						
Some college or less	Ref.	-	-	Ref.	-	-
Bachelor’s degree and higher	−0.0012	0.0017	−0.0048, 0.0022	−0.0037	0.0017	−0.0070, −0.0005 *
**Marital status**						
Married/living w partner	Ref.	-	-	Ref.	-	-
Other	0.0014	0.0024	−0.0033, 0.0061	0.0017	0.0022	−0.0028, 0.0062
Never married	−0.0044	0.0022	−0.0085, −0.0001 *	−0.0054	0.0020	−0.0094, −0.0016 *
**FPL**						
Below FPL	Ref.	-	-	Ref.	-	-
>FPL but <3× FPL	0.0035	0.0025	−0.0014, 0.0084	0.0018	0.0023	−0.0028, 0.0060
≥3× FPL	−0.0010	0.0025	−0.0060, 0.0040	−0.0048	0.0024	−0.0098, −0.0004 *
**Region**						
Northeast	0.0018	0.0024	−0.0028, 0.0066	0.0037	0.0023	−0.0007, 0.0081
Midwest	0.0008	0.0022	−0.0035, 0.0051	0.0002	0.0020	−0.0040, 0.0040
South	Ref.	-	-	Ref.	-	-
West	−0.0008	0.0021	−0.0051, 0.0034	−0.0011	0.0021	−0.0054, 0.0028
**Overall health**						
Excellent/Very Good/Good	Ref.	-	-	Ref.	-	-
Fair/Poor	0.0067	0.0026	0.0019, 0.0122 *	0.0004	0.0023	−0.0041, 0.0050
**Family experience with conditions**						
No	Ref.	-	-	Ref.	-	-
Yes	0.0058	0.0018	0.0023, 0.0095 *	−0.0017	0.0018	−0.0053, 0.0015
**COVID-19 vaccine**						
Received/intend	Ref.	-	-	Ref.	-	-
No	−0.0067	0.0021	−0.0108, −0.0023 *	−0.0087	0.0020	−0.0126, −0.0047 *
**Concern about COVID-19**						
More concerned	Ref.	-	-	Ref.	-	-
Less concerned	−0.0104	0.0018	−0.0140, −0.0069 *	−0.0078	0.0019	−0.0120, −0.0043 *
**Household members <18 years of age**	0.0014	0.0011	−0.0004, 0.0037	-	-	-
Mean squared error	0.0462	0.0034	0.0395, 0.0529	0.0336	0.0030	0.0276, 0.0396

FPL = federal poverty level. * *p* ≤ 0.05.

**Table 4:** Effects of sociodemographic variables, health experiences, attitude variables, and survey year on QALY losses using beta regression, RSV.

Variable	Mean	Bootstrap SD	95% CI	Mean	Bootstrap SD	95% CI
**Survey administration year**						
2021	Ref.	-	-	Ref.	-	-
2023	−0.0025	0.0017	−0.0059, 0.0008	−0.0007	0.0012	−0.0030, 0.0016
**Health state**						
Outpatient	−0.0051	0.0017	−0.0089, −0.0019 *	−0.0038	0.0012	−0.0064, −0.0015 *
Hospitalization	Ref.	-	-	Ref.	-	-
**Gender**						
Male	Ref.	-	-	Ref.	-	-
Female	−0.0004	0.0016	−0.0036, 0.0027	−0.0024	0.0014	−0.0056, −0.0000 *
**Age**	−0.0000	0.0001	−0.0001, 0.0001	−0.0000	0.0000	−0.00008, 0.0001
**Education**						
Some college or less	Ref.	-	-	Ref.	-	-
Bachelor’s degree and higher	−0.0022	0.0017	−0.0056, 0.0011	−0.0022	0.0013	−0.0050, 0.0001
**Marital status**						
Married/living w partner	Ref.	-	-	Ref.	-	-
Other	0.0019	0.0022	−0.0025, 0.0063	−0.0009	0.0017	−0.0043, 0.0023
Never married	−0.0014	0.0022	−0.0054, 0.0035	0.0004	0.0014	−0.0022, 0.0034
**FPL**						
Below FPL	Ref.	-	-	Ref.	-	-
>FPL but <3× FPL	−0.0013	0.0024	−0.0065, 0.0032	−0.0007	0.0017	−0.0042, 0.0024
≥3× FPL	−0.0041	0.0025	−0.0096, 0.0004	−0.0047	0.0020	−0.0091, −0.0014 *
**Region**						
Northeast	0.0008	0.0026	−0.0038, 0.0065	−0.0002	0.0017	−0.0039, 0.0030
Midwest	0.0000	0.0021	−0.0043, 0.0043	−0.0017	0.0017	−0.0054, 0.0012
South	Ref.	-	-	Ref.	-	-
West	−0.0023	0.0021	−0.0069, 0.0016	−0.0030	0.0017	−0.0070, −0.0001 *
**Overall health**						
Excellent/Very Good/Good	Ref.	-	-	Ref.	-	-
Fair/Poor	0.0034	0.0024	−0.0012, 0.0082	−0.0003	0.0017	−0.0040, 0.0030
**Family experience with conditions**						
No	Ref.	-	-	Ref.	-	-
Yes	0.0001	0.0034	−0.0054, 0.0078	−0.0046	0.0022	−0.0092, −0.0007 *
**COVID-19 vaccine**						
Received/intend	Ref.	-	-	Ref.	-	-
No	−0.0043	0.0022	−0.0088, −0.0003 *	−0.0032	0.0016	−0.0064, −0.0002 *
**COVID-19 attitudes**						
More concerned	Ref.	-	-	Ref.	-	-
Less concerned	−0.0024	0.0018	−0.0064, 0.0009	−0.0020	0.0014	−0.0053, 0.0004
**Household members <18 years of age**	0.0018	0.0011	−0.0001, 0.0042	-	-	-
Mean squared error	0.0351	0.0045	0.0263, 0.0439	0.0202	0.0035	0.0135, 0.0270

FPL = federal poverty level. * *p* ≤ 0.05. A combined marginal effects model was used to examine how the survey year and sociodemographic variables affected QALY losses associated with RSV illness.

## Data Availability

The raw data supporting the conclusions of this article will be made available by the authors upon reasonable request.

## Supplementary Materials

The following supporting information can be downloaded at https://www.mdpi.com/article/10.3390/children12020181/s1: Table S1: Health state descriptions; Table S2: Survey bid vectors; Table S3: Exclusion rules; Table S4: Respondent characteristics; Table S5: Quality-Adjusted Life Days (QALDs) lost in children and adults, 2021 survey administration, COVID-19; Table S6: Quality-Adjusted Life Days (QALDs) lost in children and adults, 2021 survey administration, RSV; Table S7: Quality-Adjusted life years (QALYs) lost, 2023 survey administration; Table S8: Quality-Adjusted Life Years (QALYs) lost due to illness in children and adults, sensitivity analyses, 2021 survey administration, COVID-19; Table S9: Quality-Adjusted Life Years (QALYs) lost due to illness in children and adults, sensitivity analyses, 2021 survey administration, RSV; Table S10: Quality-Adjusted Life Years (QALYs) lost due to illness in children and adults, stratified analyses, 2021 survey administration, COVID-19; Table S11: Effects of sociodemographic variables, health experiences, attitude variables, and survey administration year on QALY losses, using beta regression, COVID-19, adult and all frames; Table S12: Effects of sociodemographic variables, health experiences, attitude variables, and survey year on QALY losses, using beta regression, RSV, adult and all frames; Table S13: Effects of sociodemographic variables and survey administration year on QALY losses, using beta regressionCOVID-19; Table S14: Effects of sociodemographic variables and survey year on QALY losses, using beta regression, RSV; Table S15: Kolmogorov–Smirnov test comparing 2021 and 2023 survey data, QALY losses; Figure S1: Example time trade-off questions for child and spillover health states.
