# Supplementary material for: Health Preferences in Transition: Differences from Pandemic to Post-Pandemic in Valuation of COVID-19 and RSV Illness in Children and Adults
Source: Children (Basel). 2025 Jan 31;12(2):181. doi: 10.3390/children12020181 (PMC11854640; doi:10.3390/children12020181)
Supplement: Supplementary file 1 [file children-12-00181-s001.zip › children-3407636-supplementary.pdf]

Health Preferences in Transition: Differences from Pandemic to Post-Pandemic in Valuation of COVID-19 and RSV Illness in Children and Adults

Supplementary materials

Contents

Supplemental Table 1. Health state descriptions* 2

Supplemental Table 2. Survey bid vectors 6

Supplemental Table 3. Exclusion rules 7

Supplemental Table 4. Respondent characteristics 8

Supplemental Table 5. Quality-Adjusted Life Days (QALDs) lost in children and adults, 2021 survey administration, COVID-19 10

Supplemental Table 6. Quality-Adjusted Life Days (QALDs) lost in children and adults, 2021 survey administration, RSV 10

Supplemental Table 7. Quality-Adjusted Life Years (QALYs) lost, 2023 survey administration 11

Supplemental Table 8. Quality-Adjusted Life Years (QALYs) lost due to illness in children and adults, sensitivity analyses, 2021 survey administration, COVID-19 12

Supplemental Table 9. Quality-Adjusted Life Years (QALYs) lost due to illness in children and adults, sensitivity analyses, 2021 survey administration, RSV 19

Supplemental Table 10. Quality-Adjusted Life Years (QALYs) lost due to illness in children and adults, stratified analyses, 2021 survey administration, COVID-19 20

Supplemental Table 11. Effect of sociodemographic variables, health experiences, attitude variables, and survey administration year on QALY losses, by beta regression, COVID-19, adult and all frames 21

Supplemental Table 12. Effect of sociodemographic variables, health experiences, attitude variables, and survey year on QALY losses, by beta regression, RSV, adult and all frames 23

Supplemental Table 13. Effect of sociodemographic variables and survey administration year on QALY losses, by beta regression, COVID-19 25

Supplemental Table 14. RSV Effect of sociodemographic variables and survey year on QALY losses, by beta regression, RSV 29

Supplemental Table 15. Kolmogorov-Smirnov test comparing 2021 and 2023 survey administrations, QALY losses 33

Supplemental Figure 1. Example time trade off questions for child and spillover health states 34

Supplemental Table 1. Health state descriptions*

a. Child health state descriptions

Health State	Description
**Outpatient COVID-19**	Imagine that your child has an illness:<br>• Your child has a fever and cough and is very tired<br>• Your child is tested for COVID and you find out that he or she is positive<br>• You don't know when your child will get better and there is a chance that your child's illness will worsen and your child will need to be hospitalized.<br>• For the first few days, your child has difficulty doing normal activities due to his or her illness<br>• For the next week, your child continues to have a runny nose and sore throat, but is able to complete their usual activities at home<br>• For the entire time, your child is contagious to other people and must remain isolated in your home without any contact with family members and is unable to go to school or daycare<br>• Your child completely recovers and has no more problems related to having the illness.
**Hospitalized with COVID-19**	Imagine that your child has an illness:<br>• Your child has a fever and cough and is very tired.<br>• Your child has a runny nose or is congested and has a sore throat.<br>• Your child is tested for COVID-19 and you find out that he or she is positive.<br>• After about a week of not getting any better, your child starts to have problems breathing and you go to the emergency room.<br>• At the hospital your child has an X-ray and is given an IV for medications. Your child stays there for 5-10 days recovering and being treated.<br>• You don't know whether your treatment will work and there is a chance that your child's illness could worsen and that he or she would require ICU (Intensive Care Unit) care and a ventilator to help them breathe.<br>• Only one parent can stay with your child and you cannot have other visitors because your child is very contagious. All hospital staff must wear full protective clothing, masks and shields when they come into your child's room.<br>• After hospitalization, it takes a week or so for your child to return to normal activities and daycare or school.<br>• Your child completely recovers and has no more problems related to having the illness.
**Hospitalized with COVID-related multi system inflammatory syndrome (MIS-C)**	Imagine that your child has an illness:<br>• Your child develops a rash and a fever and has abdominal pain, nausea, and diarrhea.<br>• After a few days, your child is not getting better and you go to the hospital.<br>• At the hospital, your child has a chest X-ray, heart ultrasound (echo/EKG), and is treated with medications by IV.<br>• Your child continues to get worse and is admitted to the ICU (Intensive Care Unit). Your child stays there for 10 days, hooked up to the ventilator that is breathing for him or her and receiving multiple medications.<br>• After hospitalization, it takes 10 days for your child to return to normal activities.
**Chronic COVID-19 (Long COVID)**	Imagine that your child has an illness:<br>• After your child's COVID-19 illness, he or she continues to be very tired.<br>• Your child has nausea and diarrhea and has lost his or her appetite.<br>• Your child also has a rash and frequently gets headaches or other body aches.<br>• Because of your child's symptoms, he or she spends a lot of time resting and is sometimes unable to attend school or daycare.<br>• Your child's symptoms come and go. They seem to improve for a few days, but then worsen again and you don't know when your child will get better.

	• After 6 months, most children feel better, but some children continue to have symptoms for a longer period of time.
**Outpatient RSV**	Imagine that your child has an illness:<br>• Your child has a bad cough and may have wheezing.<br>• Your child has a fever, is fussy, feels tired, is not eating most of their meals, and has a runny nose.<br>• Your child is sick for a total of 10 days.<br>• For 3 days, your child has difficulty doing normal activities like playing and is unable to go to daycare or school.<br>• For 7 additional days, your child has cold symptoms like a cough and a runny nose, but is able to complete his or her usual activities.<br>• Your child completely recovers and has no more problems related to having the illness.
**Hospitalized with RSV**	Imagine that your child has an illness:<br>• Your child has a fever, feels tired, and has body aches.<br>• Your child has a cough and difficulty breathing.<br>• After a couple of days of not getting any better, your child goes to the hospital.<br>• At the hospital he or she is given an IV for fluids, oxygen to help with breathing problems, and medication for pneumonia. Your child stays in the hospital for 2-3 days, recovering and being treated.<br>• After 2-3 days in the hospital your child returns home.<br>• After hospitalization, it takes 1 more week for your child to return to normal activities like playing and attending school. Your child completely recovers and has no more problems related to having the illness.

b. Adult health state descriptions

Health State	Description
**Outpatient COVID-19**	Imagine that you have an illness:<br>• You have a moderate cough and a low-grade fever for the first few days, but are generally able to complete your usual activities despite feeling tired.<br>• You are tested for COVID-19 and find out that you are positive.<br>• You don't know when you will get better and there is a chance that your illness will worsen and you will need to be hospitalized.<br>• Over the next several days you feel extremely tired, your cough worsens, you have a consistently high fever, and severe chills. You are unable to complete normal household activities at home and are unable to go to work.<br>• For the entire time, you are contagious to other people and must remain isolated in your room without any contact with your family or friends and you cannot go to work in-person.<br>• After about 2 weeks, you are feeling better –your cough and fever are gone but you're still a bit tired. You slowly return to your usual energy level over the next couple of weeks.
**Hospitalized with COVID-19**	Imagine that you have an illness:<br>• You feel very tired and have a fever.<br>• You struggle to take a deep breath and have a bad cough, and chest pain.<br>• You are tested for COVID-19 and find out that you are positive.<br>• After about a week you are feeling worse and you go to the emergency room.<br>• At the hospital you have an X-ray and are given an IV for medications and oxygen to help you breathe more easily. You are admitted to the hospital and stay there for 5-10 days recovering and being treated.

	• You don't know whether your treatment will work and there is a chance that your illness could worsen and that you would require ICU (Intensive Care Unit) care and a ventilator to help you breathe.<br>• You are alone during most of your hospital stay. Your family cannot visit you in the hospital because you could infect them with COVID-19 and all hospital staff must wear full protective clothing, masks and shields when they come into your room to check on you.<br>• After returning home, it takes several weeks for you to return to normal activities and work.
**Hospitalized COVID-19 with ICU stay**	Imagine that you have an illness:<br>• You feel very tired and have a high fever.<br>• You have a lot of trouble breathing and a severe cough.<br>• You are tested for COVID-19 and find out that you are positive.<br>• After a couple of days of getting worse, you notice that you are so short of breath that you have trouble walking and you go to the emergency room.<br>• At the hospital you have an X-ray, are treated with several medications, and get oxygen to help you breathe more easily.<br>• You don't know whether your treatment will work and there is a chance that your illness could worsen and that you would require ICU (Intensive Care Unit) care.<br>• You continue to get worse and on your second day you are admitted to the ICU and are put on a ventilator to breathe for you. You stay there for 14 days hooked up to the ventilator, lying face down, with tubes in your stomach and bladder.<br>• Your family is told that you might not survive this illness.<br>• You are alone during most of your hospital stay. Your family cannot visit you because you are contagious. All hospital staff must wear full protective clothing, masks and shields when they come into your room to check on you.<br>• After returning home, it takes several weeks for you to return to normal activities and work.
**Chronic COVID-19 (Long COVID)**	Imagine that you have an illness:<br>• After your COVID-19 illness, you continue to be very tired and have a stuffy nose, cough, and sore throat.<br>• Your cold-like symptoms eventually improve but you remain extremely tired which makes it very difficult to work and do your normal activities.<br>• You easily become out of breath when you walk and often get dizzy when you stand up.<br>• Your joints also hurt and your brain feels foggy.<br>• Your symptoms come and go. They seem to improve for a while but then worsen again and you don't know when you'll fully recover.<br>• After 6 months, most people feel better, but some people continue to have symptoms for a longer period of time.
**Outpatient RSV**	Imagine that you have an illness:<br>• You have a bad cough, a fever, and a runny nose.<br>• You are sick for a total of 10 days.<br>• For 3 days, you have difficulty doing normal activities and are unable to go to work.<br>• For 7 additional days, you have cold symptoms like a cough and a runny nose, but are able to complete your usual activities<br>• You completely recover and have no more problems related to having the illness.
**Hospitalized with RSV**	Imagine that you have an illness:<br>• You have a fever, you feel tired, and have body aches.<br>• You have a cough and difficulty breathing.<br>• After a couple of days of not getting any better, you go to the hospital.

	• At the hospital you have an X-ray and are given an IV for fluids and oxygen to help with breathing problems. You are treated for pneumonia with medication. You stay there for 4 days recovering and being treated.<br>• After 4 days in the hospital you return home.<br>• After hospitalization, it takes 10 days for you to return to normal activities and work. You completely recover and have no more problems related to having the illness.

***** Each respondent evaluated a subset of the four health states in three question frames: (1) adult health state (≥ 18 years of age; asked the respondent to imagine how they would feel if they had the illness), (2) child health state (<18 years of age; asked the respondent to consider how *their child* would feel if *their child* had the illness), and (3) as a parent of an ill child ("spillover frame" asked the respondent to imagine how *they* would feel if *their child* had the illness). As part of the TTO task, respondents were asked how much time they would be willing to trade from the end of their life to avoid the described health state.

Supplemental Table 2. Survey bid vectors

a. Outpatient health states

	Group 1	Group 2	Group 3	Group 4
Initial bid	2 days	4 days	1 week	2 weeks
High bid	4 days	1 week	2 weeks	1 month
Low bid	1 day	2 days	4 days	1 week

b. Hospitalized and hospitalized with complications health states*

	Group 1	Group 2	Group 3	Group 4
Initial bid	2 weeks	1 month	2 months	8 months
High bid	1 month	2 months	4 months	1 year
Low bid	1 week	2 weeks	1 month	4 months

* These are combined because the effects of COVID on health-related quality of life in these two health states are expected to be similar.

c. Long COVID health states

	Group 1	Group 2	Group 3	Group 4
Initial bid	1 month	3 months	6 months	2 years
High bid	2 months	6 months	1 year	4 years
Low bid	2 weeks	1 month	3 months	1 year

Respondents were randomized to one of four groups (Groups 1-4 above) of bid vectors (selected amounts of time to trade off) for each health state (outpatient, hospitalized, hospitalized in ICU or with MIS-C, and Long COVID). Bid vectors were chosen based on prior work for health states with similar severity and evaluated for appropriateness during the pilot test and assessed during pilot testing. Respondents first answered questions using an initial bid then followed up by a second question with a higher ("High bid") or lower bid ("Low bid") based on the response to the initial bid. This was followed by a question to elicit maximum time willing to be traded. For example, respondents answering questions about outpatient COVID were first asked if they would be willing to give up 2 days from the end of their life to avoid COVID illness. If the respondent selected yes, the bid increased to 4 days. If the respondent selected no, the bid decreased to 1 day. After answering questions about these 2 preset bids, all respondents were then asked for the maximum amount of time they would be willing to give up from the end of their life to avoid the effects of the COVID illness.

Supplemental Table 3. Exclusion rules

a. Exclusion rules for primary analysis

Type	Description	Number of respondents	
		2021 survey administration	2023 survey administration
Didn't understand task	Indicated yes to the question: "I did not understand what was being asked"	3	18
Protest comments	Remove respondents who entered comments indicating that they refused to trade off time for this exercise	7	12
Poor quality responses	Entered a non-zero value for at least 3 of the 4 time categories (days, weeks, months, years) for at least 2 of 6 TTO questions (or answered 0 TTO questions.*	NA**	303
Total respondents excluded		10	327***

*Respondents were excluded when indicating overly specific timeframes (e.g., entering that they were willing to give up 2 months, 1 week, and 6 days of time from the end of their life to avoid illness)

**The 2021 survey was fielded with a different answer format and respondents could not enter TTO amounts with different units. Therefore this exclusion rule was not applied.

***Some respondents qualified for greater than 1 exclusion rule and therefore numbers do not sum.

Supplemental Table 4. Respondent characteristics

	2021 survey administration n=1014		2023 survey administration n=1186	
	No.	%	No.	%
**Gender**				
Male	501	49.4	510	43.0
Female	513	50.6	665	56.1
Other	-	-	11	0.9
**Age**				
Mean age, years	48	-	49	
Range years	18-89	-	18-93	
Ages range, years				
18-29	169	16.7	207	17.5
30-39	231	22.8	220	18.5
40-49	137	13.5	164	13.8
50-59	157	15.5	185	15.6
60-69	192	18.9	254	21.4
70-79	100	9.9	142	12.0
80+	28	2.8	14	1.2
**Race/ethnicity**				
White, non-Hispanic	641	63.8	826	69.7
Hispanic	176	17.5	123	10.4
Black, non-Hispanic	103	10.3	130	11.0
Other/mixed, non-Hispanic	84	8.4	107	9.0
**Highest level of school completed**				
Less than high school	52	5.1	31	2.6
High school diploma or GED	191	18.8	391	33.0
Some college	409	40.3	246	20.7
Bachelor’s degree	202	19.9	302	25.5
Advanced degree	160	15.8	215	18.1
**Marital status**				
Married/living with a partner	579	57.1	634	53.5
Divorced/separated/widowed	198	19.5	329	27.7
Single, never married	237	23.4	223	18.8
**Income level**				
≤ Federal poverty level (FPL)	175	17.3	296	26.4
>FPL but <3x FPL	414	40.8	347	31.0
≥3x FPL	425	41.9	477	42.6
**Children in household**				
Yes	193	19.3	404	34.1

	2021 survey n=1014		2023 survey n=1186	
	No.	%	No.	%
**Self-reported health status**				
Excellent	110	10.8	182	15.3
Very Good	398	39.3	401	33.8
Good	356	35.1	409	34.5
Fair	126	12.4	160	13.5
Poor	24	2.4	33	2.8
**Experience with COVID-19 or RSV**[a]				
COVID-19	298	29.4	703	59.3
Hospitalized with COVID-19	59	5.8	107	9.0
RSV	60	5.9	113	9.5
Hospitalized with RSV	40	3.9	55	4.6
None	665	65.6	443	37.4
**COVID-19 vaccination status/intentions**[b]				
Received/scheduled to receive	635	62.9	839	70.7
Intend to get the vaccine soon	82	8.1	43	3.6
Do not intend to get vaccine soon but may in the future	142	14.1	54	4.6
Do not intend to get vaccine	151	15.0	248	20.9
**Opinion about the risk of COVID-19: "COVID-19 is not as problematic as media presents"**[b]				
Strongly Agree	130	12.9	234	19.7
Agree	200	19.8	301	25.4
Disagree	295	29.2	404	34.1
Strongly Disagree	385	38.1	245	20.7
**Difficulty of time trade-off questions**[c]				
Easy	214	21.3	397	33.5
Somewhat Easy	326	32.4	389	32.8
Somewhat Hard	333	33.1	306	25.8
Hard	134	13.3	94	7.9
**Respondents thinking of a child of specific age**	460	45.6	632	53.3
Mean age of (theoretical) child, years	9.0	-	4.9	-
Age of (theoretical) child, range, years	0-62	-	0-59	

[a] Experience assessed with the question "Has anyone in your family ever experienced any of the following?" Respondents could have experience with more than one illness, so the percentage does not add up to 100%.

[b] Vaccination plans were assessed with the question "What have you done or are you planning to do regarding the COVID-19 vaccine?" Answer choices were dichotomized for the regression with those responding that they had already received, were scheduled to receive, or intended to get the vaccine compared with those who did not intend to get the vaccine now, but may in the future, and those who never intended to get the vaccine.

[c] Concern about COVID-19 was assessed by asking respondents if they agree with the statement "COVID-19 is not as big of a problem as the media suggests." Those who agreed or strongly agreed were termed "Less concerned about COVID" in regression analysis while those who disagreed or strongly disagreed were termed "More concerned about COVID."

FPL- Federal Poverty Level

Supplemental Table 5. Quality-Adjusted Life Days (QALDs) lost in children and adults, 2021 survey administration, COVID-19

Variable	Median	p5-p95	Mean	95% CI (bootstrapped)
**Outpatient COVID-19**				
Child	0.3	0.0 - 243.5	25.7	14.9 - 38.2
Spillover	0.1	0.0 - 26.1	13.2	5.2 - 24.0
Adult	0.2	0.0 - 16.6	6.8	2.7 - 11.9
**Hospitalized COVID-19 (no ICU)**				
Child	1.9	0.0 - 365.3	33.7	23.3 - 46.0
Spillover	0.5	0.0 - 107.4	21.8	13.8 - 31.3
Adult	1.0	0.0 - 42.1	16.4	9.2 - 25.6
**Hospitalized COVID-19 with complications**				
MIS-C (child only)				
Child	3.9	0.0 - 323.1	37.1	26.7 - 49.1
Spillover	1.2	0.0 - 199.9	29.6	18.9 - 42.1
COVID ICU (adult only)	1.4	0.0 - 60.9	16.6	9.9 - 24.6
**Long COVID**				
Child	7.0	0.0 - 313.1	40.1	29.7 - 52.2
Spillover	2.2	0.0 - 114.1	27.0	18.2 - 37.8
Adult	2.9	0.0 - 66.4	20.5	12.8 - 30.5

Supplemental Table 6. Quality-Adjusted Life Days (QALDs) lost in children and adults, 2021 survey administration, RSV

Variable	Median	p5-p95	Mean	95% CI (bootstrap)
**Outpatient RSV**				
Child	0.1	0-28.1	16.6	7.7-27.1
Spillover	0.0	0-9.6	9.1	2.8-17.5
Adult	0.0	0-4.1	6.8	1.9-12.8
**Hospitalized RSV**				
Child	0.5	0-88.4	26.5	16.2-38.5
Spillover	0.2	0-40.6	13.6	7.5-20.8
Adult	0.2	0-22.8	7.0	3.5-11.7

Supplemental Table 7. Quality-Adjusted Life Years (QALYs) lost, 2023 survey administration

a. COVID-19 illness

b. Variable	Median	5th-95th percentile	Mean	95% CI*
**QALYS lost due to outpatient COVID-19**				
Child	0.0008	0 - 0.5882	0.0651	0.044 - 0.087
Spillover	0.0002	0 - 0.2774	0.0519	0.034 - 0.072
Adult	0.0003	0 - 0.1205	0.0366	0.021 - 0.054
**QALYs lost due to hospitalized COVID-19 (no ICU)**				
Child	0.0023	0 - 0.1471	0.0391	0.026 - 0.054
Spillover	0.0004	0 - 0.1042	0.0271	0.017 - 0.039
Adult	0.0011	0 - 0.0439	0.0259	0.014 - 0.039
**QALYs lost due to hospitalized COVID-19 with complications**				
MIS-C (child only)				
Child	0.0056	0 - 0.7834	0.0885	0.066 - 0.114
Spillover	0.0018	0 - 0.4545	0.0663	0.047 - 0.089
COVID ICU (adult only)	0.0031	0 - 0.1739	0.0924	0.033 - 0.197
**QALYs lost due to Long COVID**				
Child	0.0106	0 - 0.8182	0.0940	0.072 - 0.118
Spillover	0.0034	0 - 0.4000	0.0676	0.049 - 0.088
Adult	0.0034	0 - 0.2083	0.0505	0.035 - 0.068

ICU- intensive care unit; TTO- time trade off; QALD- quality adjusted life days; QALY- quality adjusted life years

*Bootstrapped

b. RSV illness

Variable	Median	p5-p95	Mean	95% CI (bootstrap)
**QALYS lost due to outpatient RSV**				
Child	0.0004	0 - 0.6610	0.0633	0.043 - 0.086
Spillover	0.0000	0 - 0.1220	0.0371	0.022 - 0.054
Adult	0.0001	0 - 0.0721	0.0290	0.016 - 0.044
**QALYs lost due to hospitalized RSV**				
Child	0.0006	0 - 0.0606	0.0304	0.017 - 0.045
Spillover	0.0001	0 - 0.0439	0.0276	0.014 - 0.045
Adult	0.0002	0 - 0.0417	0.0259	0.014 - 0.040

Supplemental Table 8. Quality-Adjusted Life Years (QALYs) lost due to illness in children and adults, sensitivity analyses, 2021 survey administration, COVID-19

a. Excludes respondents who answered less than 50% of valuation questions

Variable	Median	Mean	p5 – p95
**QALYs lost due to outpatient COVID-19**			
Child	0.001	0.066	0.000 - 0.667
Spillover	0.000	0.037	0.000 - 0.071
Adult	0.001	0.019	0.000 - 0.046
**QALYs lost due to hospitalized COVID-19 (no ICU)**			
Child	0.005	0.088	0.000 – 1.000
Spillover	0.001	0.060	0.000 - 0.294
Adult	0.003	0.045	0.000 - 0.115
**QALYs lost due to hospitalized COVID-19 with complications**			
MIS-C (child only)			
Child	0.011	0.102	0.000 - 0.885
Spillover	0.003	0.081	0.000 - 0.547
COVID ICU (adult only)	0.004	0.046	0.000 - 0.167
**QALYs lost due to Long COVID**			
Child	0.019	0.111	0.000 - 0.857
Spillover	0.006	0.075	0.000 - 0.313
Adult	0.008	0.056	0.000 - 0.182

ICU- intensive care unit; QALY- quality adjusted life years; MIS-C- multi-system inflammatory system in children

b. Excludes respondents who provided same non-zero time trade off response across all questions (insensitive to scope)

Variable	Median	Mean	p5 – p95
**QALYs lost due to outpatient COVID-19**			
Child	0.001	0.073	0.000 -0.941
Spillover	0.000	0.036	0.000 - 0.071
Adult	0.001	0.016	0.000 - 0.046
**QALYs lost due to hospitalized COVID-19 (no ICU)**			
Child	0.006	0.089	0.000 - 0.833
Spillover	0.001	0.053	0.000 - 0.217
Adult	0.003	0.038	0.000 - 0.114
**QALYs lost due to hospitalized COVID-19 with complications**			
MIS-C (child only)			
Child	0.011	0.099	0.000 - 0.833
Spillover	0.003	0.077	0.000 - 0.385
COVID ICU (adult only)	0.004	0.040	0.000 - 0.135
**QALYs lost due to Long COVID**			
Child	0.020	0.108	0.000 - 0.769
Spillover	0.006	0.070	0.000 - 0.286
Adult	0.008	0.051	0.000 - 0.170

ICU- intensive care unit; QALY- quality adjusted life years; MIS-C- multi-system inflammatory system in children

c. Time trade off amount adjusted to life expectancy if time trade off response greater than maximum time remaining

Variable	Median	Mean	p5 – p95
**QALYs lost due to outpatient COVID-19**			
Child	0.001	0.061	0.000 - 0.667
Spillover	0.000	0.029	0.000 - 0.071
Adult	0.001	0.019	0.000 - 0.046
**QALYs lost due to hospitalized COVID-19 (no ICU)**			
Child	0.005	0.084	0.000 – 1.000
Spillover	0.001	0.058	0.000 - 0.294
Adult	0.003	0.040	0.000 - 0.115
**QALYs lost due to hospitalized COVID-19 with complications**			
MIS-C (child only)			
Child	0.011	0.096	0.000 - 0.885
Spillover	0.003	0.072	0.000 - 0.547
COVID ICU (adult only)	0.004	0.042	0.000 - 0.167
**QALYs lost due to Long COVID**			
Child	0.019	0.104	0.000 - 0.857
Spillover	0.006	0.068	0.000 - 0.313
Adult	0.008	0.050	0.000 - 0.182

ICU- intensive care unit; QALY- quality adjusted life years; MIS-C- multi-system inflammatory system in children

d. Excludes respondents who answered less than 50% of valuation questions and provided same non-zero time trade off responses across all health states. Also adjusts time trade off amounts for life expectancy.

Variable	Median	Mean	p5 – p95
**QALYs lost due to outpatient COVID-19**			
Child	0.003	0.050	0.000 - 0.139
Spillover	0.000	0.020	0.000 - 0.053
Adult	0.000	0.016	0.000 - 0.046
**QALYs lost due to hospitalized COVID-19 (no ICU)**			
Child	0.005	0.067	0.000 - 0.294
Spillover	0.001	0.043	0.000 - 0.174
Adult	0.003	0.025	0.000 - 0.077
**QALYs lost due to hospitalized COVID-19 with complications**			
MIS-C (child only)			
Child	0.011	0.082	0.000 - 0.385
Spillover	0.003	0.056	0.000 - 0.278
COVID ICU (adult only)	0.004	0.022	0.000 - 0.091
**QALYs lost due to Long COVID**			
Child	0.019	0.087	0.000 - 0.417
Spillover	0.005	0.057	0.000 - 0.278
Adult	0.008	0.032	0.000 - 0.135

ICU- intensive care unit; QALY- quality adjusted life years; MIS-C- multi-system inflammatory system in children;

e. Discounted at 3% and time trade off amounts adjusted to life expectancy if time trade off response was greater than maximum time remaining

Variable	Median	Mean	p5 – p95
**QALYs lost due to outpatient COVID-19**			
Child	0.001	0.057	0.000 – 0.627
Spillover	0.000	0.027	0.000 – 0.041
Adult	0.000	0.017	0.000 – 0.031
**QALYs lost due to hospitalized COVID-19 (no ICU)**			
Child	0.003	0.076	0.000 – 1.000
Spillover	0.001	0.053	0.000 – 0.244
Adult	0.001	0.035	0.000 – 0.080
**QALYs lost due to hospitalized COVID-19 with complications**			
MIS-C (child only)			
Child	0.006	0.082	0.000 – 0.842
Spillover	0.002	0.063	0.000 – 0.358
COVID ICU (adult only)	0.002	0.036	0.000 – 0.119
**QALYs lost due to Long COVID**			
Child	0.011	0.088	0.000– 0.842
Spillover	0.003	0.056	0.000 – 0.228
Adult	0.004	0.041	0.000 – 0.137

ICU- intensive care unit; QALY- quality adjusted life years; MIS-C- multi-system inflammatory system in children

f. Time trade off amounts adjusted for life expectancy and for worse health at end of life*

Variable	Median	Mean	p5 – p95
**QALYs lost due to outpatient COVID-19**			
Child	0.001	0.051	0.000 – 0.567
Spillover	0.000	0.025	0.000 – 0.061
Adult	0.000	0.016	0.000 – 0.039
**QALYs lost due to hospitalized COVID-19 (no ICU)**			
Child	0.004	0.071	0.000 – 0.850
Spillover	0.001	0.049	0.000 – 0.250
Adult	0.002	0.034	0.000 – 0.098
**QALYs lost due to hospitalized COVID-19 with complications**			
MIS-C (child only)			
Child	0.009	0.081	0.000 – 0.752
Spillover	0.003	0.061	0.000 – 0.465
COVID ICU (adult only)	0.003	0.036	0.000 – 0.142
**QALYs lost due to Long COVID**			
Child	0.016	0.088	0.000 – 0.729
Spillover	0.005	0.058	0.000 – 0.266
Adult	0.007	0.042	0.000 – 0.155

ICU- intensive care unit; QALY- quality adjusted life years; MIS-C- multi-system inflammatory system in children

* Uses 0.85 health utility for ages 75-89. (Fryback DG, Dunham NC, Palta M, Hanmer J, Buechner J, Cherepanov D, Herrington SA, Hays RD, Kaplan RM, Ganiats TG, Feeny D. US norms for six generic health-related quality-of-life indexes from the National Health Measurement study. Medical care. 2007 Dec 1;45(12):1162-70.)

g. Time trade off amounts adjusted for life expectancy, discounting at 3%, and worse health at end of life*

Variable	Median	Mean	p5 – p95
**QALYs lost due to outpatient COVID-19**			
Child	0.000	0.047	0.000 – 0.523
Spillover	0.000	0.023	0.000 – 0.034
Adult	0.000	0.013	0.000 – 0.026
**QALYs lost due to hospitalized COVID-19 (no ICU)**			
Child	0.003	0.063	0.000 – 0.733
Spillover	0.001	0.043	0.000 – 0.205
Adult	0.001	0.030	0.000 – 0.068
**QALYs lost due to hospitalized COVID-19 with complications**			
MIS-C (child only)			
Child	0.005	0.066	0.000 – 0.676
Spillover	0.002	0.051	0.000 – 0.283
COVID ICU (adult only)	0.002	0.031	0.000 – 0.101
**QALYs lost due to Long COVID**			
Child	0.009	0.072	0.000 – 0.676
Spillover	0.003	0.046	0.000 – 0.193
Adult	0.004	0.034	0.000 – 0.115

ICU- intensive care unit; QALY- quality adjusted life years; MIS-C- multi-system inflammatory system in children

* Uses 0.85 health utility for ages 75-89. (Fryback DG, Dunham NC, Palta M, Hanmer J, Buechner J, Cherepanov D, Herrington SA, Hays RD, Kaplan RM, Ganiats TG, Feeny D. US norms for six generic health-related quality-of-life indexes from the National Health Measurement study. Medical care. 2007 Dec 1;45(12):1162-70.)

Supplemental Table 9. Quality-Adjusted Life Years (QALYs) lost due to illness in children and adults, sensitivity analyses, 2021 survey administration, RSV

a. Excludes respondents that answered less than 50% of valuation questions

Variable	Median	Mean	p5 – p95
**QALYs lost due to outpatient RSV**			
Child	0.0004	0.032	0 - 0.063
Spillover	0.0001	0.017	0 - 0.023
Adult	0.0001	0.019	0 - 0.011
**QALYs lost due to hospitalized RSV**			
Child	0.0013	0.064	0 - 0.200
Spillover	0.0005	0.037	0 - 0.111
Adult	0.0005	0.020	0 - 0.065

b. Excludes respondents who gave the same non-zero time trade off amount across all questions

Variable	Median	Mean	p5 – p95
**QALYs lost due to outpatient RSV**			
Child	0.0003	0.046	0 - 0.077
Spillover	0.0001	0.023	0 - 0.026
Adult	0.0001	0.016	0 - 0.011
**QALYs lost due to hospitalized RSV**			
Child	0.0013	0.068	0 - 0.239
Spillover	0.0004	0.029	0 - 0.080
Adult	0.0005	0.010	0 - 0.060

c. Adjusts time trade off amount to left expectancy if time trade off response was greater than maximum time remaining

Variable	Median	Mean	p5 – p95
**QALYs lost due to outpatient RSV**			
Child	0.0004	0.0380	0.-0.0769
Spillover	0.0001	0.0203	0.-0.0263
Adult	0.0001	0.0180	0.-0.0111
**QALYs lost due to hospitalized RSV**			
Child	0.0013	0.0616	0.-0.2419
Spillover	0.0004	0.0365	0.-0.1111
Adult	0.0005	0.0193	0.-0.0625

* Uses 0.85 health utility for ages 75-89. (Fryback DG, Dunham NC, Palta M, Hanmer J, Buechner J, Cherepanov D, Herrington SA, Hays RD, Kaplan RM, Ganiats TG, Feeny D. US norms for six generic health-related quality-of-life indexes from the National Health Measurement study. Medical care. 2007 Dec 1;45(12):1162-70.)

Supplemental Table 10. Quality-Adjusted Life Years (QALYs) lost due to illness in children and adults, stratified analyses, 2021 survey administration, COVID-19

a. COVID-19 QALY loss stratified by concern about COVID-19

Variable	More concerned about COVID-19			Less concerned about COVID-19		
	Median	Mean	p5 – p95	Median	Mean	p5 – p95
**QALYs lost due to outpatient COVID-19**						
Child	0.001	0.096	0.000 - 1.000	0.001	0.014	0.000 - 0.026
Spillover	0.000	0.047	0.000 - 0.098	0.000	0.013	0.000 - 0.010
Adult	0.001	0.026	0.000 - 0.053	0.000	0.004	0.000 - 0.008
**QALYs lost due to hospitalized COVID-19 (no ICU)**						
Child	0.008	0.106	0.000 - 1.000	0.001	0.064	0.000 - 0.200
Spillover	0.002	0.081	0.000 - 0.962	0.000	0.016	0.000 - 0.039
Adult	0.005	0.064	0.000 - 0.192	0.001	0.005	0.000 - 0.024
**QALYs lost due to hospitalized COVID-19 with complications**						
MIS-C (child only)						
Child	0.017	0.092	0.000 - 0.571	0.004	0.125	0.000 - 1.000
Spillover	0.005	0.070	0.000 - 0.547	0.001	0.107	0.000 - 1.000
COVID ICU (adult only)	0.005	0.054	0.000 - 0.192	0.002	0.026	0.000 - 0.053
**QALYs lost due to Long COVID**						
Child	0.024	0.107	0.000 - 0.714	0.004	0.117	0.000 - 1.000
Spillover	0.011	0.080	0.000 - 0.370	0.001	0.061	0.000 - 0.227
Adult	0.011	0.055	0.000 - 0.216	0.001	0.058	0.000 - 0.116

ICU- intensive care unit; QALY- quality adjusted life years; MIS-C- multi-system inflammatory system in children

b. QALY loss amounts stratified by vaccination intentions

Variable	Received/intend to receive			Do not intend to receive		
	Median	Mean	p5 – p95	Mean	Median	p5 – p95
**QALYs lost due to outpatient COVID-19**						
Child	0.001	0.072	0.000 – 1.000	0.001	0.064	0.000 - 0.133
Spillover	0.000	0.041	0.000 - 0.088	0.000	0.020	0.000 - 0.044
Adult	0.001	0.022	0.000 - 0.053	0.000	0.004	0.000 - 0.009
**QALYs lost due to hospitalized COVID-19 (no ICU)**						
Child	0.007	0.086	0.000 - 0.313	0.002	0.110	0.000 - 1.000
Spillover	0.002	0.058	0.000 - 0.231	0.000	0.064	0.000 - 1.000
Adult	0.004	0.047	0.000 - 0.147	0.001	0.041	0.000 - 0.115
**QALYs lost due to hospitalized COVID-19 with complications**						
MIS-C (child only)						
Child	0.015	0.103	0.000 - 0.571	0.004	0.099	0.000 – 1.000
Spillover	0.004	0.077	0.000 - 0.351	0.002	0.092	0.000 – 1.000
COVID ICU (adult only)	0.006	0.052	0.000 - 0.167	0.001	0.032	0.000 - 0.065
**QALYs lost due to Long COVID**						
Child	0.023	0.109	0.000 - 0.556	0.010	0.113	0.000 - 1.000
Spillover	0.011	0.075	0.000 - 0.313	0.001	0.072	0.000 - 0.885
Adult	0.010	0.058	0.000 - 0.171	0.001	0.053	0.000 - 0.269

ICU- intensive care unit; QALY- quality adjusted life years; MIS-C- multi-system inflammatory system in children

Supplemental Table 11. Effect of sociodemographic variables, health experiences, attitude variables, and survey administration year on QALY losses, by beta regression, COVID-19, adult and all frames

a. Adults

Variable	Mean	Bootstrap SD	95% CI
Survey administration year			
2021	Ref.	-	
2023	-0.0042	0.0011	-0.0063, -0.0021*
Health state			
Outpatient	-0.0157	0.0019	-0.020, -0.012*
Hospitalization	-0.0078	0.0015	-0.0110, -0.0049*
ICU	-0.0026	0.0015	-0.0055, 0.0004
Long Covid	Ref.	-	
Gender			
Male	Ref.	-	
Female	-0.0000	0.0010	-0.0020, 0.0019
Age	0.0001	0.0000	0.0001, 0.0002*
Education			
Some college or less	Ref.	-	
Bachelor's degree and higher	0.0014	0.0010	-0.0005, 0.0036
Marital status			
Married/living w partner	Ref.	-	
Other	0.0019	0.0015	-0.0008, 0.0052
Never married	0.0005	0.0012	-0.0019, 0.0030
FPL			
Below FPL	Ref.	-	
>FPL but <3x FPL	0.0011	0.0016	-0.0019, 0.0044
≥ 3x FPL	-0.0012	0.0016	-0.0042, 0.0018
Region			
Northeast	0.0005	0.0014	-0.0022, 0.0032
Midwest	0.0014	0.0013	-0.0011, 0.0040
South	Ref.	-	
West	0.0003	0.0013	-0.0021, 0.0029
Overall health			
Excellent/Very good/Good	Ref.	-	
Fair/poor	0.0027	0.0016	-0.0001, 0.0062
Family experience with conditions			
No	Ref.	-	
Yes	0.0005	0.0010	-0.0017, 0.0025
COVID-19 vaccine			
Received/intend	Ref.	-	
Do not intend	-0.0052	0.0013	-0.0077, -0.0027*
COVID-19 attitudes			

More concerned	Ref.	-	
Less concerned	-0.0063	0.0011	-0.0087, -0.0042*
Mean squared error	0.0221	0.0025	0.0174, 0.0272

b. All frames

Variable	Mean	Bootstrap SD	95% CI
Survey administration year			
2021	Ref.	-	
2023	-0.0049	0.0009	-0.0066, -0.0032*
Health state			
Outpatient	-0.0229	0.0014	-0.0257, -0.0202*
Hospitalization	-0.0127	0.0013	-0.0152, -0.0102*
ICU	-0.0048	0.0017	-0.0081, -0.0015*
MIS-C	-0.0044	0.0014	-0.0072, -0.0015*
Long Covid	Ref.	-	
Frame			
Child	0.0089	0.0011	0.0068, 0.0112*
Spillover	-0.0011	0.0010	-0.0030, 0.0010
Adult	Ref.	-	
Gender			
Male	Ref.	-	
Female	0.0008	0.0008	-0.0008, 0.0024
Age	0.0002	0.0000	0.0001, 0.0002*
Education			
Some college or less	Ref.	-	
Bachelor's degree and higher	-0.0008	0.0008	-0.0024, 0.0008
Marital status			
Married/living w partner	Ref.	-	
Other	0.0016	0.0012	-0.0007, 0.0040
Never married	-0.0030	0.0010	-0.0050, -0.0010*
FPL			
Below FPL	Ref.	-	
>FPL but <3x FPL	0.0022	0.0012	-0.0001, 0.0046
$\geq$ 3x FPL	-0.0022	0.0012	-0.0046, 0.0002
Region			
Northeast	0.0019	0.0012	-0.0005, 0.0041
Midwest	0.0009	0.0010	-0.0012, 0.0028
South	Ref.	-	
West	-0.0005	0.0010	-0.0025, 0.0016
Overall health			
Excellent/Very good/Good	Ref.	-	
Fair/poor	0.0033	0.0012	0.0010, 0.0058*
Family experience with conditions			
No	Ref.	-	
Yes	0.0015	0.0009	-0.0002, 0.0031
COVID-19 vaccine			

Received/intend	Ref.	-	
No	-0.0070	0.0010	-0.0091, -0.0050*
Concern about COVID-19			
More concerned	Ref.	-	
Less concerned	-0.0082	0.0009	-0.0100, -0.0064*
Mean squared error	0.0341	0.0018	0.0307, 0.0376

FPL = federal poverty level
*p<0.05

Supplemental Table 12. Effect of sociodemographic variables, health experiences, attitude variables, and survey year on QALY losses, by beta regression, RSV, adult and all frames

a. Adults

Variable	Mean	Bootstrap SD	95% CI
Survey administration year			
2021	Ref.	-	
2023	-0.0016	0.0009	-0.0034, 0.0001
Health state			
Outpatient	-0.0046	0.0012	-0.0071, -0.0024*
Hospitalization	Ref.	-	
Gender			
Male	Ref.	-	
Female	-0.0024	0.0010	-0.0045, -0.0005*
Age	-0.0000	0.0000	-0.0001, 0.0000
Education			
Some college or less	Ref.	-	
Bachelor's degree and higher	0.0009	0.0010	-0.0010, 0.0029
Marital status			
Married/living w partner	Ref.	-	
Other	0.0011	0.0014	-0.0014, 0.0040
Never married	0.0013	0.0012	-0.0009, 0.0037
FPL			
Below FPL	Ref.	-	
>FPL but <3x FPL	0.0002	0.0014	-0.0027, 0.0027
$\geq$ 3x FPL	-0.0019	0.0015	-0.0053, 0.0007
Region			
Northeast	0.0013	0.0013	-0.0013, 0.0041
Midwest	0.0004	0.0012	-0.0019, 0.0031
South	Ref.	-	
West	-0.0020	0.0012	-0.0045, 0.0002
Overall health			
Excellent/Very good/Good	Ref.	-	
Fair/poor	0.0009	0.0014	-0.0016, 0.0039

Family experience with conditions			
No	Ref.	-	
Yes	-0.0022	0.0016	-0.0055, 0.0007
COVID-19 vaccine			
Received/intend	Ref.	-	
Do not intend	-0.0026	0.0012	-0.0052, -0.0003*
Concern about COVID-19			
More concerned	Ref.	-	
Less concerned	-0.0012	0.0010	-0.0034, 0.0007
Mean squared error	0.0163	0.0031	0.0104, 0.0221

a. All frames

Variable	Mean	Bootstrap SD	95% CI
Survey administration year			
2021	Ref.	-	
2023	-0.0016	0.0007	-0.0031, -0.0002*
Health state			
Outpatient	-0.0047	0.0008	-0.0065, -0.0032*
Hospitalization	Ref.	-	
Frame			
Child	0.0064	0.0012	0.0043, 0.0088*
Spillover	0.0003	0.0008	-0.0013, 0.0019
Adult	Ref.	-	
Gender			
Male	Ref.	-	
Female	-0.0021	0.0008	-0.0037, -0.0006*
Age	-0.0000	0.0000	-0.0001, 0.0000
Education			
Some college or less	Ref.	-	
Bachelor's degree and higher	-0.0010	0.0007	-0.0024, 0.0005
Marital status			
Married/living w partner	Ref.	-	
Other	0.0006	0.0010	-0.0014, 0.0026
Never married	0.0002	0.0009	-0.0016, 0.0022
FPL			
Below FPL	Ref.	-	
>FPL but <3x FPL	-0.0004	0.0011	-0.0026, 0.0015
≥ 3x FPL	-0.0036	0.0012	-0.0061, -0.0014*
Region			
Northeast	0.0007	0.0011	-0.0013, 0.0029
Midwest	-0.0005	0.0010	-0.0025, 0.0013
South	Ref.	-	
West	-0.0026	0.0010	-0.0047, -0.0009*
Overall health			
Excellent/Very good/Good	Ref.	-	
Fair/poor	0.0011	0.0011	-0.0010, 0.0033
Family experience with conditions			
No	Ref.	-	
Yes	-0.0024	0.0012	-0.0049, -0.0000*
COVID-19 vaccination intentions			
Received/intend	Ref.	-	
No	-0.0035	0.0010	-0.0055, -0.0016*

Concern about COVID-19			
More concerned	Ref.	-	
Less concerned	-0.0019	0.0008	-0.0036, -0.0003*
Mean squared error	0.0240	0.0022	0.0199, 0.0283

FPL = federal poverty level
*p≤0.05
A combined marginal effects model was used to examine how fielding year and sociodemographic variables affect QALY losses associated with RSV illness.

Supplemental Table 13. Effect of sociodemographic variables and survey administration year on QALY losses, by beta regression, COVID-19

a. Child

Variable	Mean	Bootstrap SD	95% CI
Survey administration year			
2021	Ref.	-	
2023	-0.0081	0.0018	-0.0116, -0.0047*
Health state			
Outpatient	-0.0299	0.0028	-0.0358, -0.0246*
Hospitalization	-0.0166	0.0026	-0.0221, -0.0118*
MIS-C	-0.0058	0.0026	-0.0109, -0.0008*
Long Covid	Ref.	-	
Gender			
Male	Ref.	-	
Female	0.0054	0.0017	0.0022, 0.0088*
Age	0.0003	0.0001	0.0002, 0.0005*
Education			
Some college or less	Ref.	-	
Bachelor's degree and higher	0.0000	0.0017	-0.0035, 0.0035
Marital status			
Married/living w partner	Ref.	-	
Other	0.0017	0.0024	-0.0028, 0.0065
Never married	-0.0039	0.0022	-0.0080, 0.0005
FPL			
Below FPL	Ref.	-	
>FPL but <3x FPL	0.0044	0.0024	-0.0005, 0.0092
≥ 3x FPL	0.0001	0.0024	-0.0047, 0.0048
Region			
Northeast	0.0031	0.0024	-0.0015, 0.0080
Midwest	0.0017	0.0022	-0.0025, 0.0060
South	Ref.	-	
West	0.0002	0.0021	-0.0041, 0.0043
Household members <18 years of age	0.0017	0.0010	-0.0002, 0.0039
Mean squared error	0.0462	0.0034	0.03952, 0.0529

b. Spillover

Variable	Mean	Bootstrap SD	95% CI
Survey administration year			
2021	Ref.	-	
2023	-0.0031	0.0016	-0.0061, -0.0000*
Health state			
Outpatient	-0.0218	0.0026	-0.0273, -0.0168*
Hospitalization	-0.0136	0.0025	-0.0189, -0.0090*
MIS-C	-0.0057	0.0023	-0.0105, -0.0012*
Long Covid	Ref.	-	
Gender			
Male	Ref.	-	
Female	0.0000	0.0015	-0.0031, 0.0030
Age	0.0002	0.0001	0.0001, 0.0003*
Education			
Some college or less	Ref.	-	
Bachelor's degree and higher	-0.0017	0.0016	-0.0049, 0.0013
Marital status			
Married/living w partner	Ref.	-	
Other	0.0022	0.0022	-0.0021, 0.0067
Never married	-0.0044	0.0020	-0.0083, -0.0004*
FPL			
Below FPL	Ref.	-	
>FPL but <3x FPL	0.0029	0.0022	-0.0015, 0.0070
≥ 3x FPL	-0.0029	0.0023	-0.0075, 0.0012
Region			
Northeast	0.0045	0.0023	0.0002, 0.0090*
Midwest	0.0007	0.0020	-0.0033, 0.0045
South	Ref.	-	
West	0.0002	0.0020	-0.0039, 0.0041
Mean squared error	0.0335	0.0030	0.0275, 0.0396

c. Adults

Variable	Mean	Bootstrap SD	95% CI
Survey administration year			
2021	Ref.	-	
2023	-0.0048	0.0010	-0.0068, -0.0028*
Health state			
Outpatient	-0.0153	0.0019	-0.0193, -0.0118*
Hospitalization	-0.0076	0.0015	-0.0108, -0.0047*
ICU	-0.0025	0.0015	-0.0054, 0.0005
Long Covid	Ref.	-	
Gender			
Male	Ref.	-	
Female	0.0007	0.0010	-0.0012, 0.0025
Age	0.0002	0.0000	0.0001, 0.0002*

Education			
Some college or less	Ref.	-	
Bachelor's degree and higher	0.0024	0.0011	0.0004, 0.0046*
Marital status			
Married/living w partner	Ref.	-	
Other	0.0025	0.0015	-0.0002, 0.0058
Never married	0.0010	0.0012	-0.0014, 0.0035
FPL			
Below FPL	Ref.	-	
>FPL but <3x FPL	0.0016	0.0015	-0.0014, 0.0045
$\geq$ 3x FPL	-0.0005	0.0014	-0.0034, 0.0022
Region			
Northeast	0.0016	0.0014	-0.0011, 0.0043
Midwest	0.0019	0.0013	-0.0006, 0.0046
South	Ref.	-	
West	0.0011	0.0013	-0.0013, 0.0037
Mean squared error	0.0221	0.0025	0.0174, 0.0271

d. All frames

Variable	Mean	Bootstrap SD	95% CI
Survey administration year			
2021	Ref.	-	
2023	-0.0053	0.0008	-0.0070, -0.0037*
Health state			
Outpatient	-0.0222	0.0014	-0.0250, -0.0195*
Hospitalization	-0.0124	0.0013	-0.0149, -0.0100*
ICU	-0.0047	0.0017	-0.0079, -0.0014*
MIS-C	-0.0044	0.0015	-0.0072, -0.0015*
Long Covid	Ref.	-	
Frame			
Child	0.0088	0.0011	0.0067, 0.0111*
Spillover	-0.0013	0.0010	-0.0033, 0.0008
Adult	Ref.	-	
Gender			
Male	Ref.	-	
Female	0.0019	0.0008	0.0003, 0.0035*
Age	0.0002	0.0000	0.0002, 0.0003*
Education			
Some college or less	Ref.	-	
Bachelor's degree and higher	0.0006	0.0008	-0.0010, 0.0022
Marital status			
Married/living w partner	Ref.	-	
Other	0.0022	0.0012	-0.0001, 0.0046
Never married	-0.0023	0.0010	-0.0043, -0.0003*
FPL			
Below FPL	Ref.	-	

>FPL but <3x FPL	0.0030	0.0012	0.0007, 0.0052*
$\geq$ 3x FPL	-0.0010	0.0012	-0.0033, 0.0013
Region			
Northeast	0.0030	0.0012	0.0007, 0.0053*
Midwest	0.0016	0.0010	-0.0005, 0.0036
South	Ref.	-	
West	0.0006	0.0010	-0.0014, 0.0027
Mean squared error	0.0341	0.0018	0.0307, 0.0376

FPL = federal poverty level

A combined marginal effects model was used to examine how fielding year and sociodemographic variables affect QALY losses associated with COVID-19 illness.

Supplemental Table 14. RSV Effect of sociodemographic variables and survey year on QALY losses, by beta regression, RSV

a. Child

Variable	Mean	Bootstrap SD	95% CI
Survey administration year			
2021	Ref.	-	
2023	-0.0026	0.0016	-0.0057, 0.0006
Health state			
Outpatient	-0.0048	0.0017	-0.0085, -0.0018*
Hospitalization	Ref.	-	
Gender			
Male	Ref.	-	
Female	-0.0004	0.0016	-0.0034, 0.0027
Age	0.0000	0.0001	-0.0001, 0.0001
Education			
Some college or less	Ref.	-	
Bachelor's degree and higher	-0.0013	0.0016	-0.0045, 0.0019
Marital status			
Married/living w partner	Ref.	-	
Other	0.0020	0.0022	-0.0021, 0.0065
Never married	-0.0006	0.0021	-0.0045, 0.0040
FPL			
Below FPL	Ref.	-	
>FPL but <3x FPL	-0.0012	0.0024	-0.0065, 0.0031
≥ 3x FPL	-0.0039	0.0024	-0.0090, 0.0005
Region			
Northeast	0.0015	0.0026	-0.0030, 0.0072
Midwest	0.0005	0.0021	-0.0036, 0.0048
South	Ref.	-	
West	-0.0016	0.0020	-0.0057, 0.0023
Household members <18 years of age	0.0018	0.0011	0.0001, 0.0042*
Mean squared error	0.0356	0.0045	0.0266, 0.0441

b. Spillover

Variable	Mean	Bootstrap SD	95% CI
Survey administration year			
2021	Ref.	-	
2023	-0.0009	0.0012	-0.0032, 0.0014
Health state			
Outpatient	-0.0037	0.0012	-0.0062, -0.0014*
Hospitalization	Ref.	-	
Gender			
Male	Ref.	-	
Female	-0.0024	0.0013	-0.0054, -0.0002*
Age	0.0000	0.0000	-0.0000, 0.0001
Education			
Some college or less	Ref.	-	
Bachelor's degree and higher	-0.0014	0.0012	-0.0040, 0.0008
Marital status			
Married/living w partner	Ref.	-	
Other	-0.0005	0.0016	-0.0035, 0.0027
Never married	0.0011	0.0014	-0.0015, 0.0041
FPL			
Below FPL	Ref.	-	
>FPL but <3x FPL	-0.0003	0.0016	-0.0037, 0.0027
≥ 3x FPL	-0.0036	0.0018	-0.0076, -0.0006*
Region			
Northeast	0.0002	0.0017	-0.0032, 0.0035
Midwest	-0.0013	0.0016	-0.0047, 0.0017
South	Ref.	-	
West	-0.0025	0.0016	-0.0061, 0.0004
Mean squared error	0.0208	0.0036	0.0139, 0.0277

c. Adults

Variable	Mean	Bootstrap SD	95% CI
Survey administration year			
2021	Ref.	-	
2023	-0.0018	0.0009	-0.0034, 0.0001
Health state			
Outpatient	-0.0045	0.0011	-0.0068, -0.0024*
Hospitalization	Ref.	-	
Gender			
Male	Ref.	-	
Female	-0.0024	0.0009	-0.0044, -0.0006*
Age	-0.0000	0.0000	-0.0001, 0.0000
Education			
Some college or less	Ref.	-	

Bachelor's degree and higher	0.0014	0.0010	-0.0005, 0.0033
Marital status			
Married/living w partner	Ref.	-	
Other	0.0012	0.0013	-0.0011, 0.0041
Never married	0.0018	0.0012	-0.0002, 0.0043
FPL			
Below FPL	Ref.	-	
>FPL but <3x FPL	0.0004	0.0013	-0.0025, 0.0028
≥ 3x FPL	-0.0014	0.0014	-0.0044, 0.0009
Region			
Northeast	0.0017	0.0013	-0.0009, 0.0044
Midwest	0.0007	0.0012	-0.0015, 0.0034
South	Ref.	-	
West	-0.0016	0.0011	-0.0038, 0.0006
Mean squared error	0.0162	0.0031	0.0100, 0.0219

d. All frames

Variable	Mean	Bootstrap SD	95% CI
Survey administration year			
2021	Ref.	-	
2023	-0.0017	0.0007	-0.0032, -0.0003*
Health state			
Outpatient	-0.0046	0.0008	-0.0063, -0.0031*
Hospitalization	Ref.	-	
Frame			
Child	0.0063	0.0011	0.0043, 0.0088*
Spillover	0.0003	0.0008	-0.0013, 0.0020
Adult	Ref.	-	
Gender			
Male	Ref.	-	
Female	-0.0021	0.0007	-0.0036, -0.0010*
Age	-0.0000	0.0000	-0.0000, 0.0000
Education			
Some college or less	Ref.	-	
Bachelor's degree and higher	-0.0002	0.0007	-0.0016, 0.0012
Marital status			
Married/living w partner	Ref.	-	
Other	0.0008	0.0010	-0.0011, 0.0027
Never married	0.0009	0.0009	-0.0009, 0.0028
FPL			
Below FPL	Ref.	-	
>FPL but <3x FPL	-0.0002	0.0010	-0.0024, 0.0018
≥ 3x FPL	-0.0030	0.0011	-0.0053, -0.0009*
Region			
Northeast	0.0013	0.0011	-0.0008, 0.0035
Midwest	-0.0001	0.0010	-0.0020, 0.0018

South	Ref.	-	
West	-0.0020	0.0009	-0.0040, -0.0003*
Mean squared error	0.0243	0.0022	0.0202, 0.0286

FPL = federal poverty level

*$p \leq 0.05$

Supplemental Table 15. Kolmogorov-Smirnov test comparing 2021 and 2023 survey administrations, QALY losses

Variable	Null hypothesis	Child		Spillover		Adult	
		D-statistic	P-value	D-statistic	P-value	D-statistic	P-value
**QALYs lost due to outpatient COVID-19**	2021 contains smaller values than 2023	0.047	0.458	0.043	0.529	0.066	0.227
	2021 contains larger values than 2023	-0.165	0.000*	-0.184	0.000*	-0.090	0.064
	Distributions are the same	0.165	0.000*	0.184	0.000*	0.090	0.128
**QALYs lost due to hospitalized COVID-19**	2021 contains smaller values than 2023	0.000	1.000	0.000	1.000	0.000	1.000
	2021 contains larger values than 2023	-0.162	0.000*	-0.197	0.000*	-0.120	0.007*
	Distributions are the same	0.162	0.000*	0.197	0.000*	0.120	0.014*
**QALYs lost due to hospitalized COVID-19 with complications**	2021 contains smaller values than 2023	0.013	0.947	0.035	0.666	0.011	0.963
	2021 contains larger values than 2023	-0.152	0.001*	-0.170	0.000*	-0.102	0.035*
	Distributions are the same	0.152	0.001*	0.170	0.000*	0.102	0.069
**QALYs lost due to Long COVID**	2021 contains smaller values than 2023	0.005	0.992	0.010	0.967	0.013	0.945
	2021 contains larger values than 2023	-0.173	0.000*	-0.163	0.000*	-0.103	0.031*
	Distributions are the same	0.173	0.000*	0.163	0.000*	0.103	0.063
**QALYs lost due to outpatient RSV**	2021 contains smaller values than 2023	0.104	0.025	0.090	0.068	0.075	0.148
	2021 contains larger values than 2023	-0.117	0.009*	-0.099	0.037*	-0.077	0.133
	Distributions are the same	0.117	0.018*	0.099	0.074	0.077	0.265
**QALYs lost due to hospitalized RSV**	2021 contains smaller values than 2023	0.002	0.998	0.021	0.862	0.016	0.919
	2021 contains larger values than 2023	-0.175	0.000*	-0.204	0.000*	-0.139	0.001*
	Distributions are the same	0.175	0.000*	0.204	0.000*	0.139	0.003*

*p<0.05

Supplemental Figure 1. Example time trade off questions for child and spillover health states

Figure 1a. Child

Outpatient COVID-19

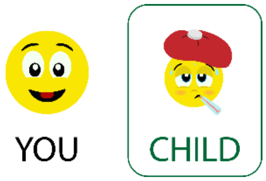

Imagine that your child has an illness:

- Your child has a fever and cough and is very tired
- Your child is tested for COVID-19 and you find out that he or she is positive
- You don't know when your child will get better and there is a chance that your child's illness will worsen and your child will need to be hospitalized.
- For the first few days, your child has difficulty doing normal activities due to his or her illness
- For the next week, your child continues to have a runny nose and sore throat, but is able to complete his or her usual activities at home.
- For the entire time, your child is contagious to other people and must remain isolated in his or her room without any contact with family members and is unable to go to school or daycare
- Your child completely recovers and has no more problems related to having the illness.

Imagine that you have 41 years left to live.

What portion of your life, if any, would you have been willing to give up from the end of YOUR life to avoid your child's experience with the illness? You can choose any amount of time in days, weeks, months, or years.

Would you be willing to give up 2 weeks in order to avoid this scenario?

- ○ Yes
- ○ No

Figure 1b. Spillover

Outpatient COVID-19

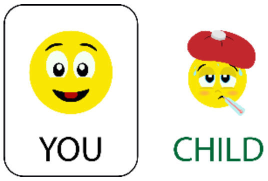

Imagine that your child has an illness:

- Your child has a fever and cough and is very tired.
- Your child is tested for COVID-19 and you find out that he or she is positive.
- You don't know when your child will get better and there is a chance that your child's illness will worsen and your child will need to be hospitalized.
- For the first few days, your child has difficulty doing normal activities due to his or her illness.
- For the next week, your child continues to have a runny nose and sore throat, but is able to complete their usual activities at home.
- For the entire time, your child is contagious to other people and must remain isolated in his or her room without any contact with family members and is unable to go to school or daycare.
- Your child completely recovers and has no more problems related to having the illness.

Imagine that you have 41 years left to live.

What portion of your life, if any, would you have been willing to give up from the end of YOUR life to avoid the effects of the illness on YOU as your child's parent? Your child would continue to have the illness, and you would continue to care for him or her, but YOU would not be affected in any way by your child's condition. You can choose any amount of time in days, weeks, months, or years.

Would you be willing to give up 2 weeks in order to avoid this scenario?

- ○ Yes
- ○ No

Figure 1c. Adult

Outpatient COVID-19

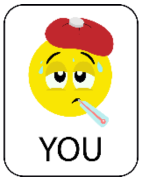

Imagine that you have an illness:

- You have a moderate cough and a low-grade fever for the first few days, but are generally able to complete your usual activities despite feeling tired.

- You are tested for COVID-19 and find out that you are positive.
- You don't know when you will get better and there is a chance that your illness will worsen and you will need to be hospitalized.
- Over the next several days you feel extremely tired, your cough worsens, you have a consistently high fever, and severe chills. You are unable to complete normal household activities at home and are unable to go to work.
- For the entire time, you are contagious to other people and must remain isolated in your room without any contact with your family or friends and you cannot go to work in-person.

After about 2 weeks, you are feeling better –your cough and fever are gone but you're still a bit tired. You slowly return to your usual energy level over the next couple of weeks.

Imagine that you have 41 years left to live.

What portion of your life, if any, would you have been willing to give up from the end of <u>YOUR</u> life to avoid your child's experience with the illness? You can choose any amount of time in days, weeks, months, or years.

Would you be willing to give up 2 weeks in order to avoid this scenario?

- o Yes
- o No
